# Unveiling Alternative Splicing in HPV-Negative HNSCC: Links to Tumorigenesis and Immune Landscape

**DOI:** 10.1016/j.identj.2025.103857

**Published:** 2025-12-15

**Authors:** Shensui Li, Mianmian Duan, Xiangrui Ma, Yadong Wu, Jianyong Kang, Zhenglong Tang, Xudong Tian

**Affiliations:** a Department of Oral and Maxillofacial Surgery, Guizhou Medical University, Guiyang, Guizhou, China; b Department of Stomatology, The Second Affiliated Hospital of Guizhou University of Traditional Chinese Medicine, Guiyang, Guizhou, China; c Department of Oral and Maxillofacial Surgery, Binzhou Medical University Hospital, Binzhou, Shandong, China

**Keywords:** Alternative splicing, Head and neck squamous cell carcinoma, Human papillomavirus negative, Prognosis, Immune microenvironment

## Abstract

**Background:**

Alternative splicing (AS) plays crucial roles in tumorigenesis by regulating cancer development, progression, and patient outcomes. However, the prognostic value of AS in HPV-negative head and neck squamous cell carcinoma (HNSCC), a subtype with distinct clinical features and poor prognosis compared to HPV-positive HNSCC, remains underexplored. This study aimed to develop a robust predictive signature based on AS events in HPV-negative HNSCC.

**Methods:**

We retrieved RNA-sequencing (RNA-seq) data and clinical profiles of the HNSCC cohort from The Cancer Genome Atlas (TCGA) via its data portal. Percentage Spliced In (PSI) values were derived from TCGA SpliceSeq. To identify prognostic AS events in HPV-negative HNSCC, we first performed univariate Cox analysis. Subsequently, we used Least Absolute Shrinkage and Selection Operator (LASSO) regression and stepwise multivariate Cox regression to develop a prognostic signature. The efficacy of this signature in predicting outcomes was assessed using Kaplan-Meier survival curves and receiver operating characteristic (ROC) analyses. Additionally, gene set enrichment analysis (GSEA) was applied to the prognostic signatures. The levels of tumor-infiltrating immune cells were quantified using established deconvolution algorithms (e.g., CIBERSORT, xCell, MCP-counter).

**Results:**

We identified 42,849 AS events across 388 HPV-negative HNSCC cases, of which 1,062 events were significantly associated with patient prognosis. We developed a prognostic signature based on the 21 most significant AS events, demonstrating high accuracy in predicting survival in HPV-negative HNSCC. This signature emerged as an independent prognostic factor. GSEA and immune microenvironment profiling revealed that the low-risk group exhibited enrichment in immune activation and antigen presentation pathways, in contrast to the high-risk group, which was linked to cell proliferation processes.

**Conclusions:**

We successfully constructed and validated a novel AS-related prognostic signature that enhances personalized survival prediction in patients with HPV-negative HNSCC**.**

## Introduction

Head and neck squamous cell carcinoma (HNSCC) is the sixth most common cancer worldwide, with squamous cell carcinoma accounting for over 90% of cases.^1^ Traditional risk factors include tobacco, alcohol, and betel nut quid consumption, while human papilloma virus (HPV) infection has emerged as a significant etiological factor.^2^^,^^3^ HPV status serves as a critical molecular and clinical classifier in HNSCC, dividing cases into 2 distinct entities: HPV-positive and HPV-negative HNSCC.^4^^,^^5^ HPV-negative HNSCC, which accounts for approximately 80% of cases, typically presents with distinct molecular alterations, including TP53 mutations, and demonstrates more aggressive clinical behavior.^1^^,^^6^^,^^7^ These tumors often show poorer differentiation, higher rates of lymph node metastasis, and increased resistance to conventional therapies compared to their HPV-positive counterparts.^8^^,^^9^ Despite advances in diagnosis and multimodal therapeutic strategies, the 5-year survival rate for HPV-negative HNSCC remains notably low (approximately 50%), significantly worse than the 80% survival rate observed in HPV-positive cases.^10^^,^^11^ This marked disparity in outcomes underscores the urgent need to identify novel and specific biomarkers for HPV-negative HNSCC to enhance our understanding of disease progression mechanisms and improve diagnostic and therapeutic strategies.

Alternative splicing (AS) represents a regulated process during gene expression, and their dysregulation can lead to the production of aberrant mature transcripts, potentially triggering tumor initiation and progression.^10^^,^^12^^,^^13^ AS refers to a regulated process in eukaryotic cells where a single gene produces multiple different mature mRNAs through variable splicing of pre-mRNA, ultimately resulting in the translation of distinct proteins.^14^ Almost all phenotypic characteristics of tumor cells are influenced by AS changes, including metabolism, apoptosis, cell cycle control, invasion, metastasis, and angiogenesis.^14^^,^^15^ Previous studies have demonstrated that AS event signatures in HNSCC could serve as independent prognostic factors.^16^^,^^17^ However, these studies did not stratify their analyses by HPV status. Given the distinct clinical, pathological, and molecular characteristics between HPV-positive and HPV-negative HNSCC, along with the significantly worse prognosis of HPV-negative cases,^1^^,^^10^^,^^18^ focused investigation of AS events in HPV-negative HNSCC is warranted.

Although TCGA datasets facilitate AS analysis in cancer, studies focused on HPV-negative HNSCC remain scarce. There are very few reports on the correlation between AS events and the clinical outcomes of HPV-negative HNSCC patients. In this study, a comprehensive analysis was performed by using the datasets of The Cancer Genome Atlas (TCGA), TCGA Splice-Seq and Broad GDAC Firehose to discuss the prognostic value of AS events in patients with HPV-negative HNSCC patients. Our findings may contribute to develop new biomarkers for HPV-negative HNSCC patients.

## Methods

**Fig. 1:**
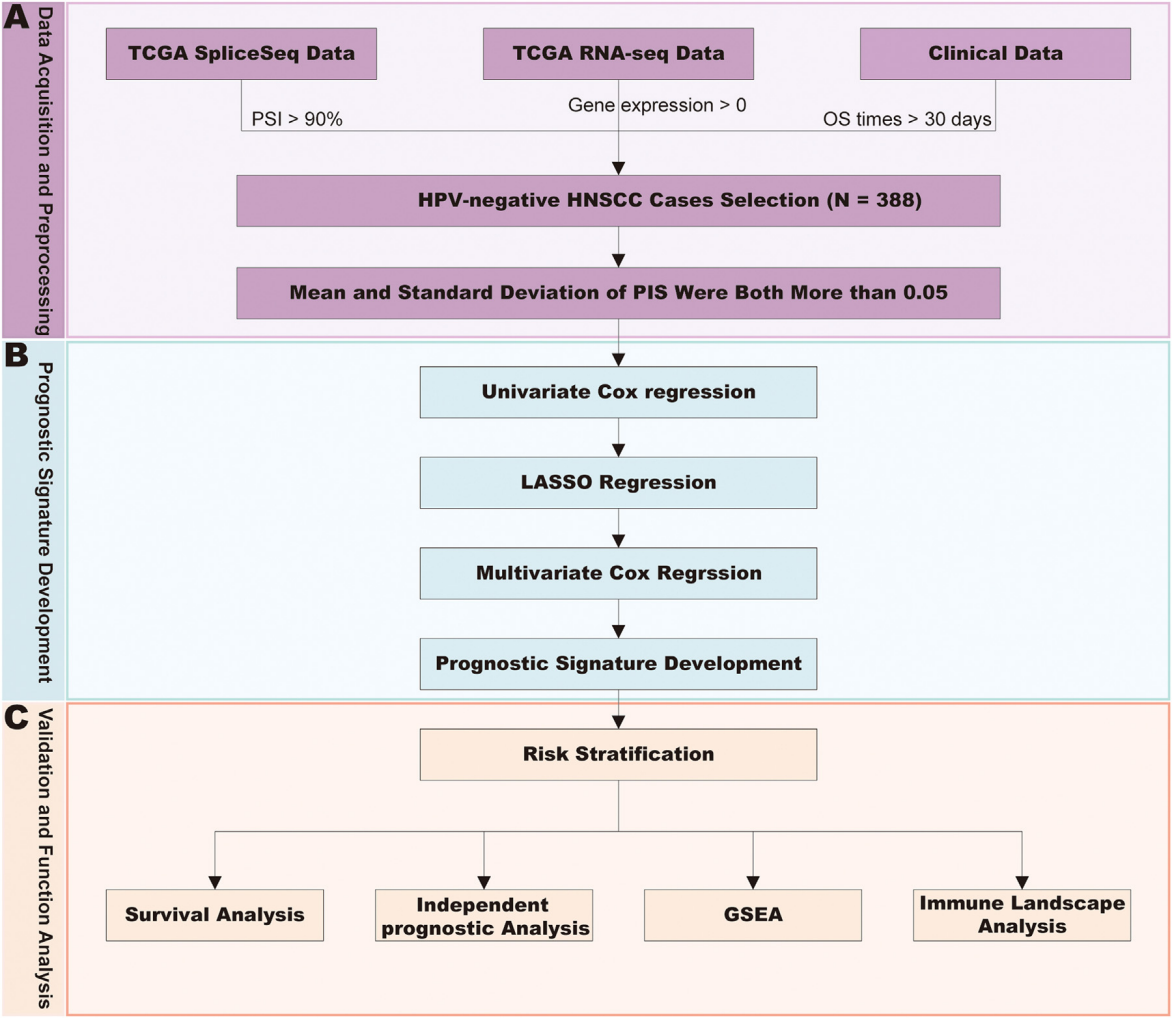
Overview of the study design and analysis workflow. (A) Data Acquisition and Preprocessing: We obtained TCGA SpliceSeq data (requiring PSI > 90%), TCGA RNA-seq data (gene expression > 0), and corresponding clinical data (OS > 30 days) from HPV-negative HNSCC cases (N = 388). Only cases with both mean and standard deviation of PSI > 0.05 were retained for subsequent analyses. (B) Prognostic Signature Development: Candidate variables were first screened by univariate Cox regression, followed by LASSO regression for feature selection, resulting in 21 AS events. The final prognostic signature was established through multivariate Cox regression. (C) Validation and Function Analysis: The predictive performance of the signature was evaluated using Kaplan-Meier survival analyses and ROC curve analyses. Functional characterization was performed through Gene Set Enrichment Analysis (GSEA) and immune landscape analyses using the CIBERSORT, xCell, and MCP-counter methods.

To enhance clarity for readers, we present our methodology in a systematic workflow (Figure 1). Our analysis consisted of 3 major steps: Data acquisition and preprocessing, prognostic signature development, validation and function analysis.

### Data acquisition and preprocessing

**Fig. 2:**
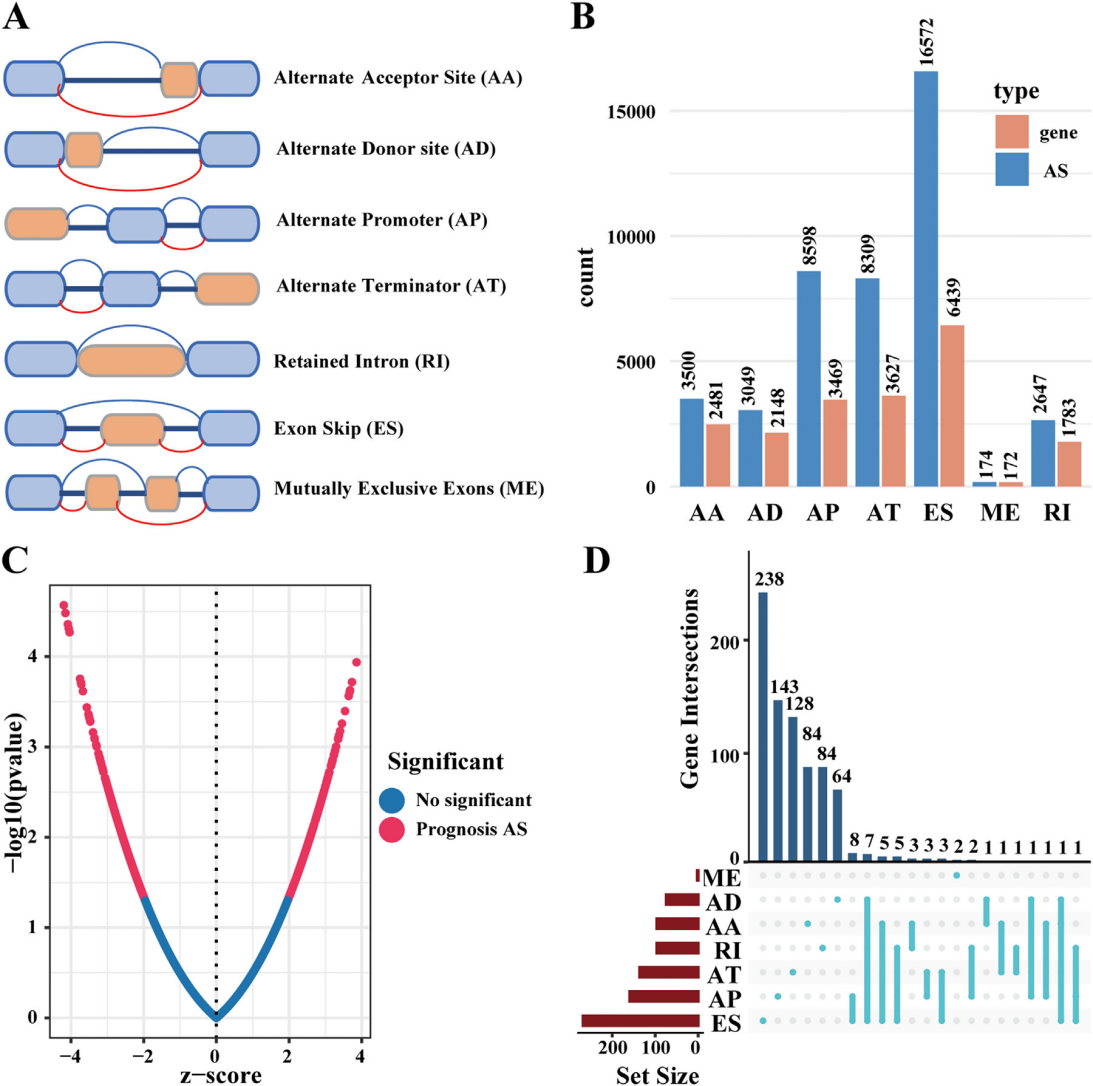
Overview of AS events in TCGA HPV-negative HNSCC cohort. (A) Seven types of AS events are illustrated including alternate acceptor site (AA), alternate donor site (AD), alternate promoter (AP), alternate terminator (AT), retained intron (RI), exon skip (ES), and mutually exclusive exons (ME). (B) The number of AS events and their corresponding genes for each AS type in 388 HPV-negative HNSCC patients, involving a total of 42,849 AS events. (C) The distribution of prognosis-related AS events revealed by volcano plot, identifying 1,062 survival-related AS events (*P* < 0.05). (D) The upset plot showing the complex interactions and overlap among different types of survival-related AS events, illustrating how single genes can undergo multiple types of alternative splicing events associated with prognosis.

We retrieved RNA sequencing data of the HNSCC cohort from The Cancer Genome Atlas (TCGA) via its data portal using the 'TCGAbiolinks' R package.^19^ For gene annotation, transcript names were mapped to gene symbols, and only genes with mean expression values greater than zero were retained. Alternative splicing event profiles were downloaded from TCGA SpliceSeq (https://bioinformatics.mdanderson.org).^20^ The Percent spliced In (PSI) value, ranging from 0 to 1, was used to quantify AS events.^17^ We only included samples with PSI values greater than 90%. Seven distinct types of AS events were analyzed (Figure 2A): alternate acceptor sites (AA), alternate donor sites (AD), alternate promoters (AP), alternate terminators (AT), retained introns (RI), exon skips (ES), and mutually exclusive exons (ME). Clinical data was obtained from the Broad GDAC Firehose database (https://gdac.broadinstitute.org). From the clinical data, we selected samples that were HPV-negative and had overall survival (OS) times greater than 30 days (Figure 1A).

### Identification of survival: associated AS events and development of the prognostic signature

We used the 'impute' R package to handle missing AS PSI values.^21^ The AS events whose sample mean and standard deviation of PSI were both more than 0.05 were retained.^22^ To identify survival-associated AS events, we performed univariate Cox regression analysis with a significance threshold of *P* < 0.05. Subsequently, we employed LASSO regression to screen for significant prognostic AS events, followed by stepwise multivariate Cox regression analysis to obtain β values (regression coefficients) for each prognostic AS event in the model (Figure 1B). The risk score was calculated using the following formula: Risk Score = β1*PSI1 + β2*PSI2 + ... + βn*PSIn.

### Validation and function analysis

To evaluate the prognostic significance of our signature, the patients were stratified into high-risk and low-risk groups based on the median risk score. We conducted Kaplan-Meier survival analysis to compare outcomes between these groups with differences assessed using the *log-rank test*. We generated risk curves, scatterplots, and expression heatmaps after reordering samples according to risk scores.

We performed univariate Cox regression analysis incorporating the risk scores and clinical variables. Factors with *P* < 0.05 were included in subsequent multivariate Cox regression analysis to identify independent prognostic factors. We assessed the predictive performance using receiver operating characteristic (ROC) curves and calculated area under curve (AUC) values.

We performed differential expression analysis between high-risk and low-risk groups using the 'DESeq2′ R package. Gene set enrichment analysis (GSEA) was conducted using the "clusterProfiler" R package^23^ with Gene Ontology (GO) gene sets from the "msigdbr" R package. The top 20 most significant pathways were selected for visualization.

To characterize the immune cell infiltration patterns between high-risk and low-risk groups, we employed 3 well-established computational methods: CIBERSORT,^24^ xCell,^25^ and MCP-counter.^26^ These algorithms were used to quantify tumor-infiltrating immune cell levels across the entire cohort. Differences in immune cell infiltration levels between groups were assessed using the *Wilcoxon rank-sum test*, and the most significant differences were visualized (Figure 1C).

### Statistical analyses

All statistical analyses were performed using R software version 4.1.0. Continuous variables between high-risk and low-risk groups were compared using the *Wilcoxon test*. Survival analysis was conducted using Kaplan-Meier methods, with differences between groups assessed by the *Log-rank rank-sum test*. Statistical significance was defined as *P* < 0.05 for all analyses.

## Results

### Overview of AS events in HPV-negative HNSCC

The analysis of 388 HPV-negative HNSCC cases from the TCGA dataset revealed 42,849 AS events across 10,123 genes. The distribution of these events included 3500 AA events in 2481 genes, 3049 AD events in 2148 genes, 8598 AP events in 3469 genes, 8309 AT events in 3627 genes, 16,572 ES events in 6439 genes, 174 ME events in 172 genes, and 2647 RI events in 1783 genes (Figure 2B). Exon skip (ES) events were the most prevalent, while mutually exclusive exons (ME) were the least common. Notably, the number of AS events substantially exceeded the number of genes, indicating that multiple alternative splicing types frequently occur within the same gene.

### Identification of Survival: associated AS Events and Development of the Prognostic Signature

**Fig. 3:**
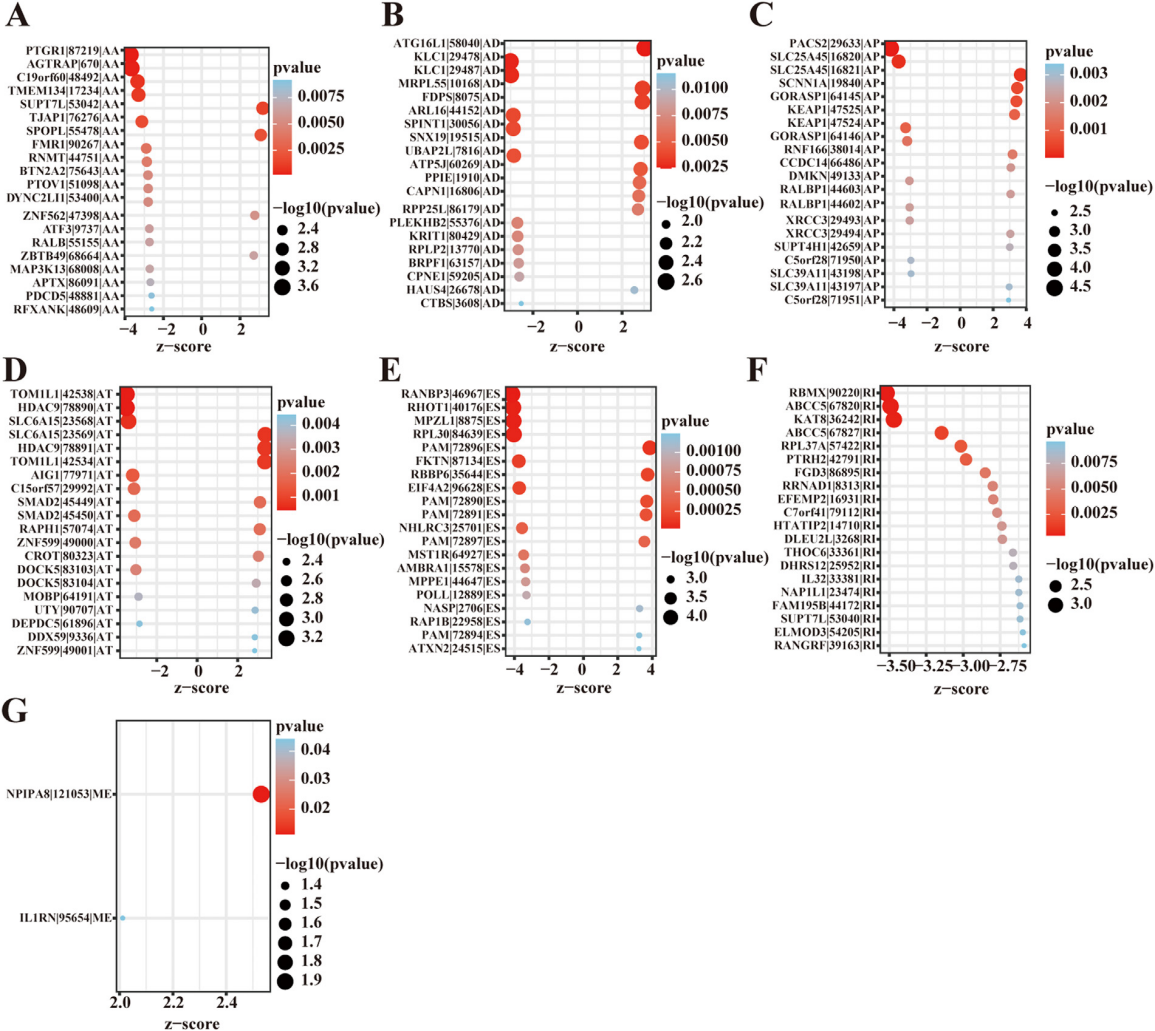
Survival‐associated AS events in HPV-negative HNSCC cohort. Forest plots showing the hazard ratios (HR) and 95% confidence intervals (CI) of the top 20 significantly survival‐related AS events for each splicing type (ME only included 2 events) (A-G). All events shown had *P* < 0.05 in univariate Cox regression analysis.

Univariate survival analysis identified 1,062 survival-related AS events (*P* < 0.05) involving 786 genes (Figure 2C and Supplementary Table 1). The distribution of these survival-related AS events is depicted in Figure 2D, where we selected the top 20 most significant AS events for each type (excluding ME) based on univariate Cox analysis (Figure 3).

**Fig. 4:**
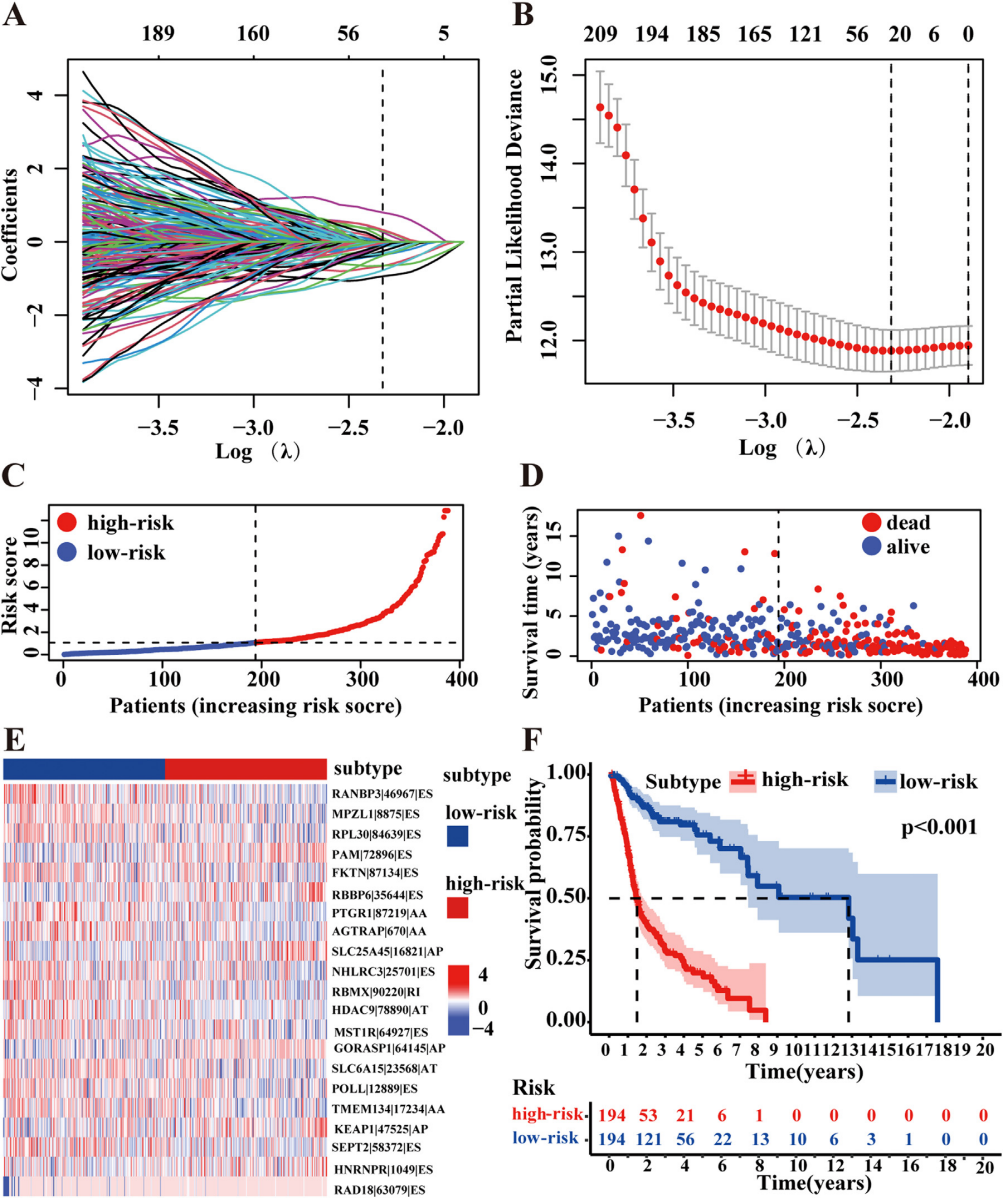
Establishment and evaluation of prognostic risk model for HPV-negative HNSCC. (A) LASSO coefficient profiles of the candidate survival‐related AS events. A coefficient profile plot is produced against the log(λ) sequence with optimal λ value selected at log(λ) = 2.31. (B) Dotted vertical lines were drawn at the optimal values by using the minimum criteria. (C) Distribution of risk scores calculated based on the 21-AS event signature. (D) The survival status and duration of HPV-negative HNSCC patients. (E) Distribution of the AS events shown by heatmap in HPV-negative HNSCC cohort. (F) Kaplan–Meier survival curves of the final prognostic predictor constructed by all types of AS events for HPV-negative HNSCC patients (stratified by median risk score = 1.07, *P* < 0.001). The blue line indicates low-risk group whereas red line indicates high-risk group.

LASSO regression analysis identified optimal parameters at log(λ) = 2.31, yielding 25 candidate AS events (Figure 4A,B, and Supplementary Table 2). Further refinement through multivariate Cox regression identified 21 survival-related AS events for our final prognostic signature. Seven events (PAM|72890|ES, RBBP6|35644|ES, SLC25A45|16821|AP, GORASP1|64145|AP, KEAP1|47525|AP, HNRNPR|1049|ES, RAD18|63079|ES) with hazard ratios >1 were classified as potential oncogenic factors, while the remaining 14 AS events with hazard ratios <1 were identified as potential tumor suppressors (Supplementary Table 2).

Based on these 21 AS events, we calculated risk scores for each patient using the formula described in Methods. Patients were stratified into high-risk and low-risk groups using the median risk score value (1.07) as the threshold. The distribution of risk scores and OS status is illustrated in Figures 4C and 4D, with the expression pattern of prognostic AS events shown in Figure 4E. Survival analysis demonstrated significantly shorter OS in the high-risk group compared to the low-risk group (*P* < 0.001) (Figure 4F).

### Validation of the AS-based prognostic signature as an independent indicator

**Fig. 5:**
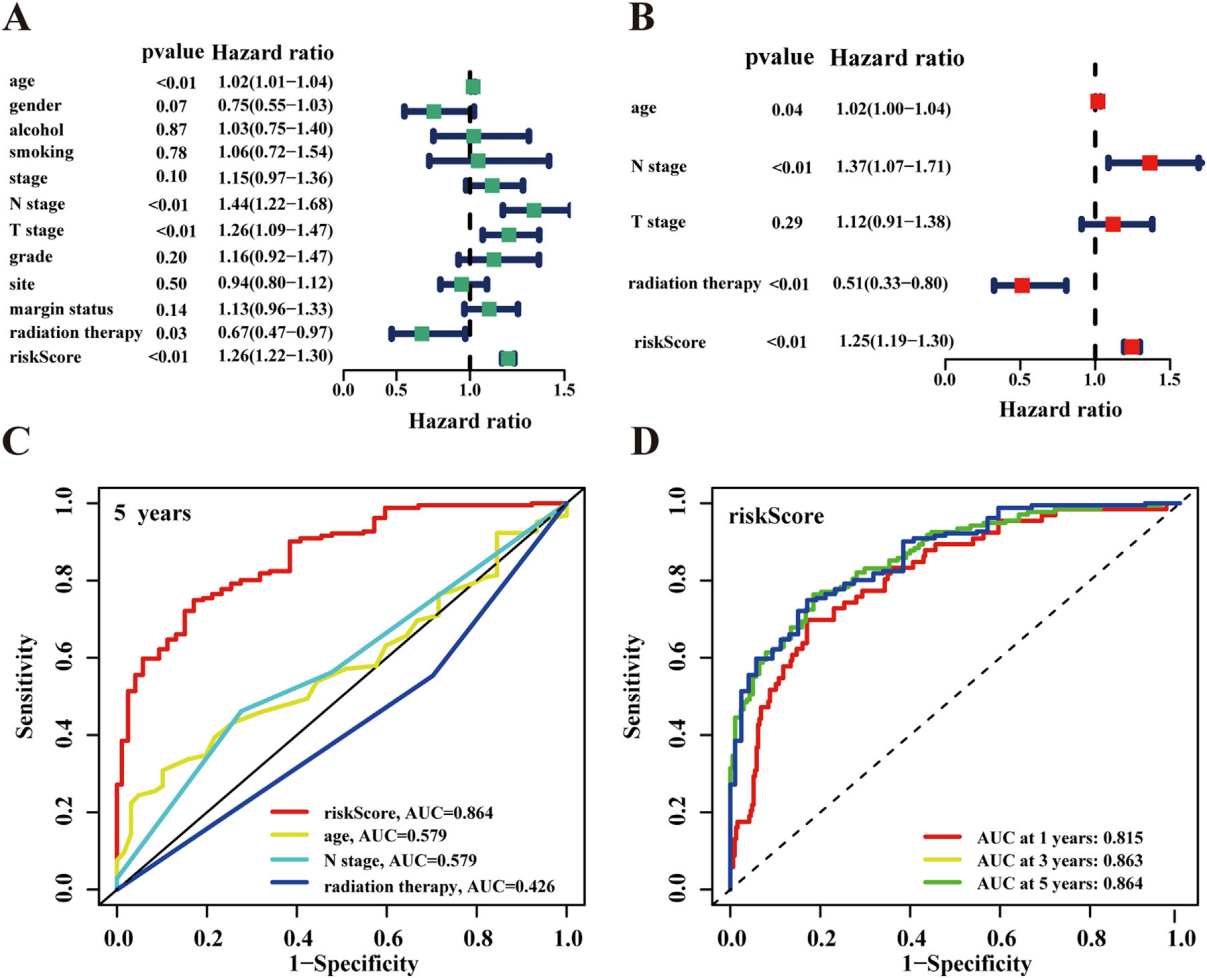
Screening and evaluation of independent prognostic factors for HPV-negative HNSCC patients. (A) Univariate analysis reveals the clinicopathological factors that are strongly correlated with overall survival (OS) in the HPV-negative HNSCC cohort, including age (*P* < 0.01, *HR* = 1.02, 95% *CI* = 1.01-1.04), N stage (*P* < 0.01, *HR* = 1.44, 95% *CI* = 1.22-1.68), T stage (*P* < 0.01, *HR* = 1.26, 95% *CI* = 1.09-1.47), radiation therapy (*P* = 0.03, *HR* = 0.67, 95% *CI* = 0.47-0.97), and risk score (*P* < 0.01, *HR* = 1.26, 95% *CI* = 1.22-1.30). (B) Multivariate analysis shows that the age, N stage, radiation therapy and risk score are the independent indicators for HPV-negative HNSCC patients. (C) ROC curves comparing the predictive performance of risk score with other clinical indicators for 5-year survival prediction. (D) ROC curves to predict the sensitivity and specificity of 1-, 3-, and 5-year survival according to the prognostic signatures, with AUC values demonstrating the model's predictive accuracy.

Univariate analysis identified several significant prognostic factors: age (*P* < 0.01, *HR* = 1.02, 95% *CI* = 1.01-1.04), N stage (*P* < 0.01, *HR* = 1.44, 95% *CI* = 1.22-1.68), T stage (*P* < 0.01, *HR* = 1.26, 95% *CI* = 1.09-1.47), radiation therapy (*P* = 0.03, *HR* = 0.67, 95% *CI* = 0.47-0.97), and risk score *(P* < 0.01, *HR* = 1.26, 95% *CI* = 1.22-1.30) (Figure 5A). Subsequent multivariate analysis confirmed age (*P* = 0.04, *HR* = 1.02, 95% *CI* = 1.00-1.04), N stage (*P* < 0.01, *HR* = 1.37, 95% *CI* = 1.07-1.71), radiation therapy (*P* < 0.01, *HR* = 0.51, 95% *CI* = 0.33-0.80), and risk score (*P* < 0.01, *HR* = 1.25, 95% *CI* = 1.19-1.30) as independent prognostic indicators (Figure 5B). ROC curve analysis for 1-, 3-, and 5-year OS demonstrated superior predictive performance of our risk signature compared to other clinical indicators (Figures 5C and 5D).

### Molecular and immunological characterization of risk groups through GSEA and immune landscape analysis

**Fig. 6:**
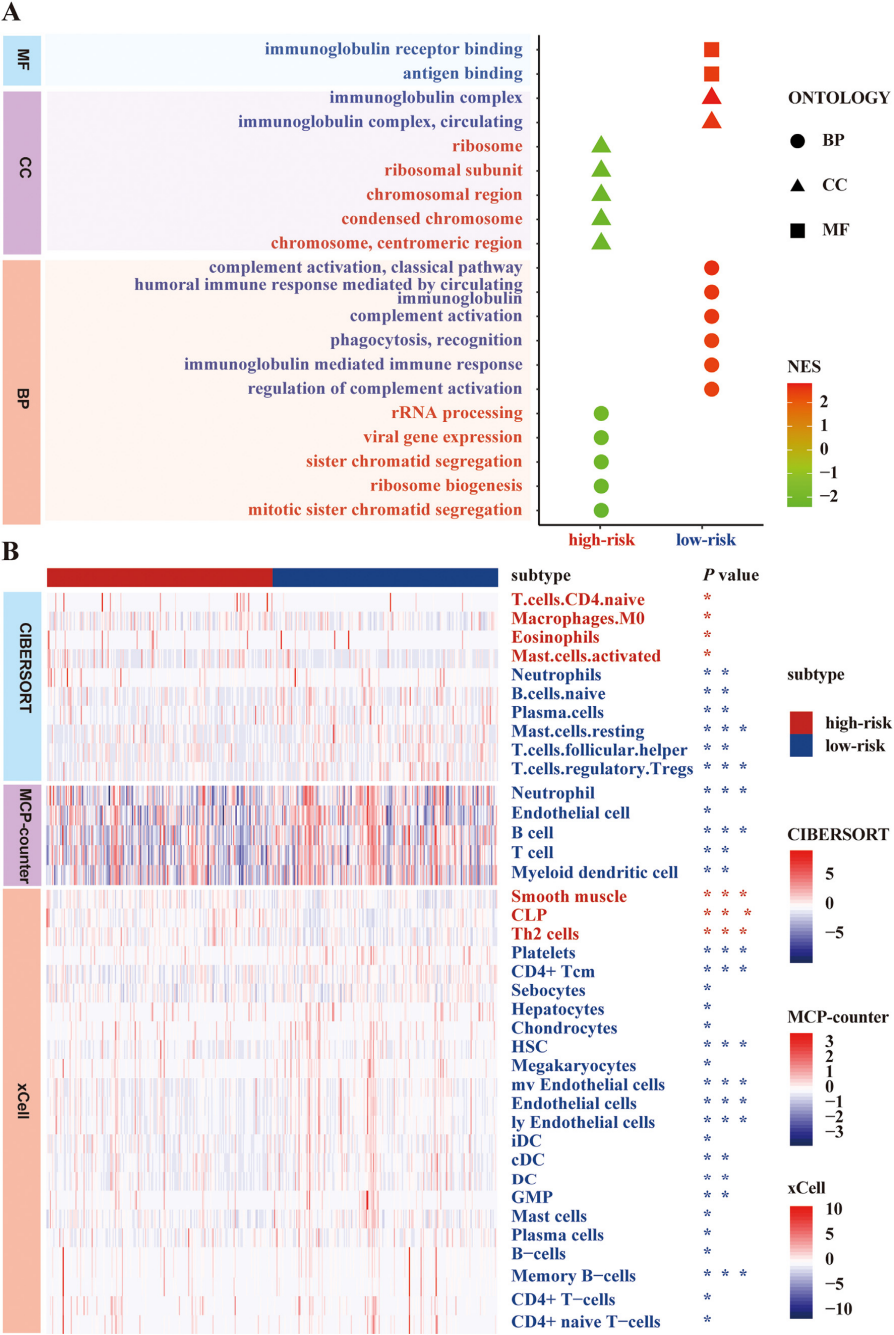
Exploring the biological characterization of the prognostic model for HPV-negative HNSCC patients. (A) The top 20 significant GSEA results in the HPV-negative HNSCC cohort are displayed, where red fonts indicate pathways enriched in the high-risk group (related to cell cycle) and blue fonts indicate pathways enriched in the low-risk group (related to immune activation). (B) Distribution of significantly different immune cells shown by heatmap in HPV-negative HNSCC cohort, analyzed using CIBERSORT, xCell, and MCP-counter algorithms. Significant statistical differences between the 2 subgroups were assessed using the *Wilcoxon test* with *P* values indicated as follows: *P < 0.05; ^⁎⁎^*P* < 0.01; ^⁎⁎⁎^*P* < 0.001.

GSEA revealed distinct biological processes between risk groups. The low-risk group showed enrichment in immune-related pathways, including complement activation, antibody-mediated immune responses, and antigen binding. Conversely, the high-risk group demonstrated enrichment in cell cycle-related processes such as mitotic sister chromatid segregation, viral gene expression, and ribosome biogenesis (Figure 6A and Supplementary Table 3).

Analysis of the tumor immune landscape showed significantly higher levels of immune cells in the low-risk group, including B cells, T cells, neutrophils, endothelial cells, and dendritic cells, particularly those associated with immune activation and antigen presentation (Figure 6B and Supplementary Table 4).

## Discussion

Head and neck squamous cell carcinoma (HNSCC) is a common and aggressive malignancy originating from squamous epithelium.^27^ Although the OS rate of HNSCC has modestly improved over the past 3 decades, subgroup analyses reveal that such improvement is largely attributable to the recognition and better outcomes of HPV-positive patients—with their markedly better prognosis—rather than to advances in multimodal treatments per se.^27^^,^^28^ In fact, end result analyses incorporating tissue-based HPV assessment have demonstrated improved survival in HPV-positive, but not in HPV-negative HNSCCs.^27^^,^^29^ Therefore, identifying novel biomarkers is essential to enhance clinical outcomes in HPV-negative HNSCC patients.^17^

Recent evidence indicates that dysregulation of alternative splicing (AS) can lead to splicing defects that generate cancer-specific markers and neoantigens.^17^^,^^30^ For example, an analysis of over 8,000 tumors across 32 cancer types identified thousands of splicing variants absent in non-malignant tissues, underscoring the potential of AS events as molecular markers in cancer diagnosis and treatment.^30^ Moreover, several studies have characterized AS profiles and developed prognostic signatures in head and neck cancers. Zhao et al. constructed a robust prognostic signature based on 5 survival-related AS events, and another study proposed an 11‐event model that convincingly predicted long‐term survival outcomes in HNSCC.^17^^,^^31^ In addition, Li et al. performed a comprehensive genome-wide analysis of the AS landscape in HNSCC, revealing novel AS events associated with carcinogenesis and the immune microenvironment, with implications for both prognosis and therapeutic responses.^32^ Similarly, Ding et al. developed multiple splicing prognostic signatures—including one integrating all 7 AS types—highlighting the prognostic value of AS events and splicing factors (SFs).^33^, ^34^, ^35^ While previous research has examined specific splice variants in HPV-negative HNSCC, such as DOCK5 and LOXL2 variants,^10^^,^^36^ comprehensive evaluation of splicing events in this subset has been limited. Our study addresses this gap by providing an in-depth analysis of AS events in HPV-negative HNSCC, offering insights into genetic diversity and molecular mechanisms underlying tumorigenesis.

In the present study, we screened for significant survival-related AS events using TCGA datasets and subsequently developed a prognostic signature by applying LASSO regression and multivariate Cox regression analyses. This signature effectively stratified HPV-negative HNSCC patients into high- and low-risk groups, with the low-risk group exhibiting significantly longer OS. Moreover, both univariate and multivariate analyses confirmed that the AS-based prognostic signature is an independent predictor of OS and demonstrates superior predictive accuracy compared to traditional TNM staging. These results underscore the potential utility of AS events as robust prognostic biomarkers for HPV-negative HNSCC.

In our cohort of 388 HPV-negative HNSCC patients, we identified 1,062 survival-related AS events across 786 genes. Among the various AS types, exon skipping (ES) was the most prevalent event, followed by alternate terminators (AT) and alternate promoters (AP). Notably, ES events are more amenable to validation via PCR compared to other AS types that require more complex primer design.^34^ By integrating all 7 types of AS events, we constructed a comprehensive prognostic signature encompassing key events in genes such as RANBP3, MPZL1, RPL30, PAM, FKTN, RBBP6, PTGR1, AGTRAP, SLC25A45, NHLRC3, RBMX, HDAC9, MST1R, GORASP1, SLC6A15, POLL, TMEM134, KEAP1, SEPT2, HNRNPR, and RAD18. Several of these genes have established roles in HNSCC pathogenesis; for example, RPL30 has been implicated in promoting invasion and metastasis in oral squamous cell carcinoma,^37^ HDAC9 overexpression is associated with increased tumor growth and cell cycle progression,^38^ and KEAP1 inhibition sensitizes HNSCC cells to ionizing radiation by impairing non-homologous end joining and inducing autophagy.^39^ Furthermore, reduced RBMX expression may serve as a favorable prognostic biomarker,^40^ whereas SEPT2 overexpression has been linked to increased cisplatin resistance.^41^

To further elucidate the biological mechanisms underlying the divergent prognoses observed between the high- and low-risk groups, we conducted GSEA in conjunction with an evaluation of the tumor immune microenvironment. Our GSEA results revealed that the low-risk group is significantly enriched in immune activation pathways, whereas the high-risk group predominantly exhibits enrichment in pathways related to cell cycle regulation and mitosis. Consistently, immune profiling indicated that the low-risk group harbors markedly higher levels of immune activating cells and antigen presenting cells (APCs), including B cells, T cells, neutrophils, endothelial cells, and dendritic cells, compared to the high-risk group. A robust presence of immune activating cells is known to suppress the growth of both primary and abscopal tumors, inhibit metastatic spread, and reduce tumor recurrence.^42^^,^^43^ In parallel, enhanced immunogenicity and improved antigen processing can mitigate tumor immune escape.^44^^,^^45^ A substantial body of evidence further supports that dense infiltration by T cells, especially cytotoxic CD8⁺ T cells, is associated with favorable clinical outcomes.^46^ Although naive B cells can differentiate into memory B cells and plasma cells upon antigen stimulation via both T cell-independent and T cell-dependent pathways,^47^ the precise role of plasma cells in the tumor milieu remains under active debate.^48^ Moreover, regulatory T cells—central to maintaining immune homeostasis—have been implicated in tumor immune evasion, with high Treg infiltration correlating with poorer prognosis in HNSCC.^49^ Similarly, an increased proportion of M0 macrophages is linked to adverse outcomes.^50^ Mast cells, which are recruited early in tumorigenesis and contribute to angiogenesis and tissue remodeling, have been associated with elevated neovascularization, increased expression of vascular endothelial growth factor and fibroblast growth factor-2 (FGF-2), and overall tumor aggressiveness.^51^^,^^52^ Additionally, tumor-induced CCL22 production by eosinophils may facilitate the recruitment of regulatory T cells, further establishing a microenvironment favorable to tumor metastasis.^53^ Although the role of neutrophils in cancer is multifaceted—with evidence supporting both pro- and anti-tumor effects effects depending on the neutrophil phenotype (N1 vs N2)—the higher neutrophil levels in the low-risk group may reflect an anti-tumor N1-like phenotype, though further investigation is needed to clarify their functional state in our cohort.^54^ Collectively, these findings suggest that multiple, interrelated mechanisms of immune escape and alterations in immune cell subtypes contribute to the progression of HPV-negative HNSCC. This study underscores the importance of comprehensively evaluating AS events not only as prognostic biomarkers but also as indicators of the underlying immune landscape in HPV-negative HNSCC.

However, several limitations of our study should be acknowledged. First, the AS-based prognostic signature requires validation in independent external cohorts and through experimental studies. Second, although numerous AS events were identified, the regulatory interactions among these events and other genes remain unclear. Third, the clinical application of AS-based signatures is currently hampered by the high cost and technical constraints of high-throughput RNA-seq. Future advancements in sequencing technologies may facilitate the translation of these prognostic features into clinical practice. Finally, the clinical implications of these potential biomarkers and therapeutic targets warrant further investigation in functional studies and clinical trials.

## Conclusions

Our study has successfully developed and validated a novel AS-related prognostic signature that enhances individualized survival prediction in HPV-negative HNSCC patients. This signature not only provides prognostic value but also offers insights into the underlying biological mechanisms, particularly immune system involvement, in HPV-negative HNSCC progression.

## Data availability

The dataset used and/or analyzed during the current study are available in the TCGA database (https://portal.gdc.cancer.gov/) and from the corresponding author upon request.

## Ethics approval and consent to participate

Not applicable.

## Consent for publication

Not Applicable.

## Conflict of interest

None disclosed.

## Appendix Supplementary materials

Image, application 1

Image, application 2

Image, application 3

Image, application 4

## Footnotes

Supplementary material associated with this article can be found in the online version at doi:10.1016/j.identj.2025.103857.
